# Can a Semi-Active Energy Harvesting Shock Absorber Mimic a Given Vehicle Passive Suspension?

**DOI:** 10.3390/s21134378

**Published:** 2021-06-26

**Authors:** Jorge A. Reyes-Avendaño, Ciro Moreno-Ramírez, Carlos Gijón-Rivera, Hugo G. Gonzalez-Hernandez, José Luis Olazagoitia

**Affiliations:** 1 School of Engineering and Sciences, Tecnologico de Monterrey, Monterrey 64849, NL, Mexico; jareyesa@tec.mx (J.A.R.-A.); crgijon@tec.mx (C.G.-R.); hgonz@tec.mx (H.G.G.-H.); 2 Industrial Engineering and Automotive Department, Universidad Antonio de Nebrija, Pirineos 55, 28040 Madrid, Spain; cmorenora@nebrija.es

**Keywords:** energy harvesting shock absorber, regenerative damper, semi-active suspension

## Abstract

Energy harvesting shock absorbers (EHSA) have made great progress in recent years, although there are still no commercial solutions for this technology. This paper addresses the question of whether, and under what conditions, an EHSA can completely replace a conventional one. In this way, any conventional suspension could be replicated at will, while recovering part of the wasted energy. This paper focuses on mimicking the original passive damper behavior by continuously varying the electrical parameters of the regenerative damper. For this study, a typical ball-screw EHSA is chosen, and its equivalent suspension parameters are tried to be matched to the initial damper. The methodology proposes several electrical control circuits that optimize the dynamic behavior of the regenerative damper from the continuous variation of a load resistance. The results show that, given a target damper curve, the regenerative damper can adequately replicate it when there is a minimum velocity in the damper. However, when the damper velocity is close to zero, the only way to compensate for inertia is through the introduction of external energy to the system.

## 1. Introduction

The future of mobility has meant that energy recovery has become more relevant in response to the growing demand for greater protection of the environment, improved air quality and optimization of resources. In the automotive industry, suspension systems represent an excellent opportunity in energy recovery. The suspension system is responsible for the characteristics of comfort and maneuverability in driving.

Vehicle suspension systems are a crucial part of vehicle safety, as they provide two important basic features: isolation from uneven terrain (comfort) and ensuring tire contact with the road, allowing vehicles to maneuver, brake and turn (safety). Shock absorbers (usually passive) allow us to damp undesired oscillations by providing viscosity to the system, for example, by passing a viscous fluid through controlled size holes. In this way, the oscillatory mechanical movement that reaches the suspension (mechanical energy) is converted into energy in the form of heat that is wasted.

There are many types of suspensions. The most common are passive suspensions, although active or energy-recovery suspensions can also be found. In this paper, the focus is on the latter, the so-called Energy Harvesting Shock Absorbers (EHSA) [1]. Energy-recovering suspensions can function as semi-active suspensions, so that it is possible to regulate the stiffness and damping coefficients of the suspension by taking advantage of the recovered energy [2].

The design of vehicle shock absorbers is determined by the vehicle manufacturers, based on the desired dynamic and comfort characteristics [3]. Usually the force-velocity (*F*-*v*) curve of the shock absorber is specified from design by the Original Equipment Manufacturer (OEM) after dynamic studies and simulations. This ideal (*F*-*v*) curve is then passed along to the shock absorber manufacturers who must adapt the design and the mechanical fluid characteristics of the passive shock absorber, both in the extension and compression movement. In this way, the shock absorber can provide the desired characteristics to the vehicle dynamics. Thus, once a vehicle is designed by the OEM, its suspension characteristics are fixed for optimum vehicle performance.

This article raises the question of whether it is possible to safely replace a given passive suspension with an energy recovery system that provides the same initial damping characteristics. In addition, the system must be able to recover some of the energy wasted by the original suspension. It is important to ensure that the new shock absorber is as close as possible to the original dynamic characteristics set by the OEM, so that the dynamic behavior of the vehicle does not change substantially.

As will be seen in the literature review that follows, various energy recovery systems and technologies for shock absorbers have been presented. Some of them propose the optimization of these systems to harvest as much energy as possible, with the best possible comfort.

Regarding regenerative shock absorbers and their optimal control, several approaches can be found in the literature. In [4], a LMI-based strategy was applied to achieve ride comfort and maximum energy harvesting simultaneously to a full car model, and an actively governed linear electrical motor was used as a shock absorber. An optimized energy conversion design of a motor-pump for regenerative shock absorber was depicted in [5], the design considers no affecting the damping control properties and it was experimentally validated using a prototype. Some reviews have been published on regenerative shock absorbers, for example, in [6], an analysis of electrical circuits and control algorithms to maximize the power output and to deliver ride comfort is depicted; high compatibility with the vehicle is identified as a future trend. This paper focus on the compatibility of the ball-screw damper as a shock absorber with respect to a commercial damper. An optimization technique is proposed to find conditions for better mimicking the commercial damper, in this context, no traditional optimal control technique is applied, as it is being considered for future work.

The issue of the feasibility of replacing a given passive suspension with another energy recuperator that ensures a dynamic behavior similar to the one replaced, so as not to alter the dynamic behavior of the vehicle for which the original suspension was designed, is not directly addressed.

On a practical level, with regard to energy efficiency, it is very interesting to be able to move vehicle shock absorbers from a merely passive, energy-dissipating function to one that takes advantage of the mechanical energy that reaches it by making it useful. These EHSAs have been extensively studied in recent years. They can be quickly classified by the type of technology used to transform the linear oscillatory motion in the suspension into recovered energy. Piezoelectric [7], hydraulic [8] and electromagnetic [9,10] systems can be found.

The most studied EHSA systems in the literature are based on electromagnetic technology which, in turn, can be of linear [11,12] and rotational [13,14] type. Within the latter, many systems have been presented to convert linear motion into rotational motion to take advantage of the rotation of a motor generator to recover energy. It is possible to find EHSA based on ball-screws [15,16], rack and pinion [17,18], hydraulic turbine [19], cable transmission [2], or based on special mechanisms [10,20].

The developments of these EHSAs have followed disparate paths, so that the performance evaluation of these EHSAs has been uneven. That is, each author has presented operating and performance results that are difficult to compare with those of other investigations. In this regard, some articles have been published that attempt to provide a review of the EHSAs presented to date [14,21] and, on the other hand, some articles propose different methodologies for evaluating their performance [22,23,24].

One of the most interesting features of these EHSAs is that it is possible to alter the electrical characteristics of the system, which allows the characteristic damping curve of the shock absorber to be changed easily. In this way, the shock absorber equipped with an EHSA cannot only recover energy, but also alter the dynamic behavior of the suspension at will. EHSAs with this feature can be considered as semi-active or active dampers, depending on the case.

The advantage of semi-active suspensions [25] is that the force applied to the wheel is directly proportional to the wheel acceleration. The disadvantage is that it is very hard to estimate the exact wheel velocities and thus the forces at any given time. In active suspensions [26], the force applied to the wheels is directly proportional to the wheel velocity, which is much easier to estimate (since it is a known quantity and only needs to be measured). However, active suspensions can only react to the wheel velocity, whereas semi-active suspensions can apply a force to the wheels due to their acceleration.

From a comfort point of view, it is interesting to be able to change the characteristic curve of the shock absorber at will, so that it can be adapted to the user’s driving preferences or the characteristics of the terrain. In some vehicle models, it is possible to change the behavior of the vehicle suspension by means of active or semi-active dampers. Most commonly, these systems allow acting on the damping of the system through the introduction of external energy to the system. For example, magnetorheological dampers [27], which incorporate magnetic particles suspended in a viscous liquid, can alter their rheological properties by the application of an external magnetic field [28,29]. In other cases, the application of external energy to a motor acting on the suspension allows the appropriate damping characteristic to be achieved [30]. Recently, studies on energy recovery dampers have shown that it is possible to change the comfort characteristics by varying the electrical parameters of the generator [13].

After this quick review of the state of the art of EHSA systems, it can be concluded that most of the bibliographic references found refer to the simulation, design or application of these energy recovery systems, focusing on their feasibility, design, functionality and performance. In some cases, the system is optimized in such a way that the recovered energy and the perceived comfort are optimized at the same time. However, there are doubts as to how this technology can be made viable and functional in existing vehicles. On the one hand, it is necessary to make efforts to reduce the price of these recovery systems, still far above the cost of the usual passive dampers. On the other hand, it is not clear which technology is suitable in each case and how its design can be optimized.

Furthermore, no references have been found that address and study in detail the question posed in this paper: Can a passive damper be replaced by an energy recovery damper while respecting its damping characteristic curve? This question can be addressed more broadly, if at the design stage, the question of what would be the optimal EHSA system to achieve a given damping curve for a given vehicle is addressed.

Therefore, this article presents the first study on the possibility of designing an energy recovery shock absorber in such a way that it mainly and primarily respects the damping characteristic curve of a given shock absorber. This study is important in the first place because, if successful, it would allow the direct replacement of passive dampers by EHSAs, as they would provide the basic dynamic and comfort functionality of the original suspension system. Secondarily, once the basic functionality of the shock absorber is assured, the nature of the recoil shock absorber makes useful a mechanical energy that would otherwise be dissipated and lost as heat.

The novelty of the present work lies therefore in the study of the design of an energy recovery shock absorber system (EHSA) that allows to functionally replicate the dynamic behavior of a passive shock absorber, through the possibility of actively acting on its operating parameters by electronically varying a resistance (real or apparent) that depends on the state of the suspension. The aim of this study is to shed light on the technical possibilities offered by EHSA systems to actively modify their operating characteristics in order to replicate a specific curve of a real shock absorber as a matter of priority, while allowing energy recovery in the process.

It is important to note that the present work is focused in the study of the setting capabilities of the EHSA system to mimic the behavior of a conventional damper. Thus, the different approaches studied do not consider the energy recovery component of the system. This part of the system will be addressed in a future work, once the dynamic capabilities of the system are demonstrated in the present research.

The rest of this work has been structured as follows. First, Section 2 presents a description of the chosen suspension system, including the associated electrical system, its equations and functional model. Based in the mathematical model of the system developed in this section, Section 3 analyzes the different force’s components appearing in the system and compares its response to a real damper unit tested in a damper test machine. Optimal values of the load resistor are found to emulate the force–velocity curve obtained for the real damper. These optimal values of the load resistor are used in Section 4 to explore different electrical approaches that allow us to replicate the real damper behavior. Finally, in Section 5, a discussion of the results obtained is made and future lines of research are presented.

## 2. System Description and Modeling

In order to model the dynamics of a suspension, only a quarter car is commonly used, meaning that only one set of components associated with the suspension is considered. Using this quarter car model allows us to focus on the analyses related to comfort and safety before considering a complete car behavior. In this case, a quarter car model is described including a ball-screw semi-active suspension acting both as a damper and as a harvester.

### 2.1. Quarter-Car Model

**Figure 1:**
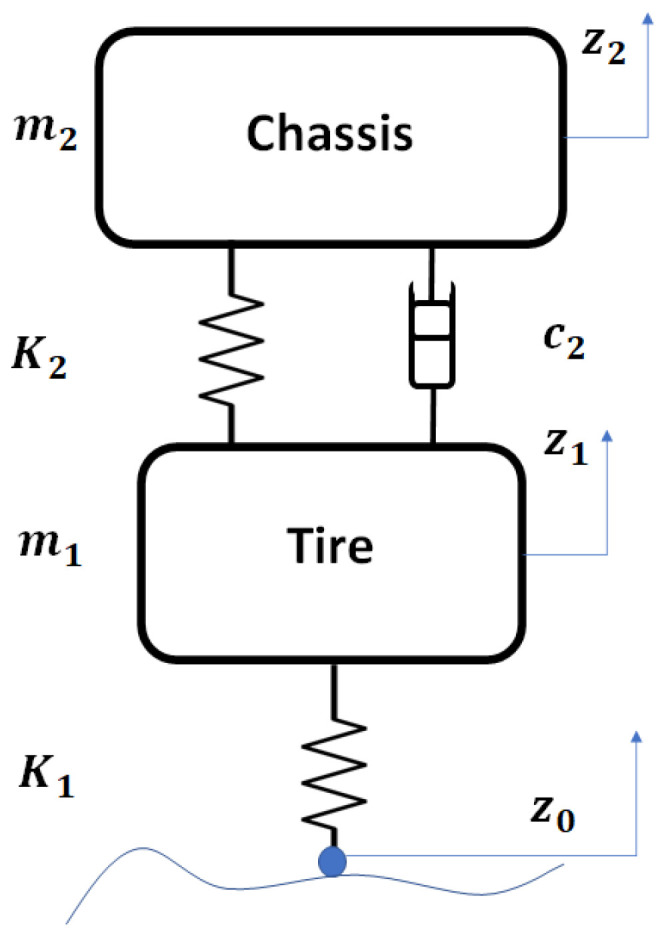
Quarter car model with Energy Harvester Shock Absorber (EHSA) ball-screw.

A typical quarter car model is a good approximation to examine the performance of a ball-screw device as automotive suspension with one degree of freedom. In Figure 1, a quarter car model with Energy Harvester Shock Absorber (EHSA) is shown, a sprung mass is denoted by $m_{1}$ representing the tire mass, which is attached to an unsprung mass $m_{2}$ representing the chassis mass.

Through this model, we can say that the function of the shock absorber is ble to isolate the vehicle chassis from unwanted vibrations transmitted by road irregularities. Conventional hydraulic shock absorbers are widely used, and the force–velocity profiles are known to ensure a balance between comfort and maneuverability. According to this profile, we will select the appropriate ball-screw parameters to ensure a very similar behavior between both systems (hydraulic suspension and ball-screw).

The dynamics of the mechanical system shown in Figure 1 can be described as:

(1) $$\begin{array}{ccc} m_{1}{\ddot{z}}_{1}-K_{1}\left(z_{1}-z_{0}\right)+K_{2}\left(z_{2}-z_{1}\right)+c_{2}\left({\dot{z}}_{2}-{\dot{z}}_{1}\right) & = & 0 \end{array}$$

(2) $$\begin{array}{ccc} m_{2}{\ddot{z}}_{2}-K_{2}\left(z_{2}-z_{1}\right)-c_{2}\left({\dot{z}}_{2}-{\dot{z}}_{1}\right) & = & 0 \end{array}$$

### 2.2. Ball-Screw Energy Harvesting Shock Absorber (BS-EHSA)

According to [21], the most popular energy conversion mechanism in automotive suspensions is the ball-screw transmission, which has a high mechanical efficiency with low power consumption, just below the rack-pinion system.

**Figure 2:**
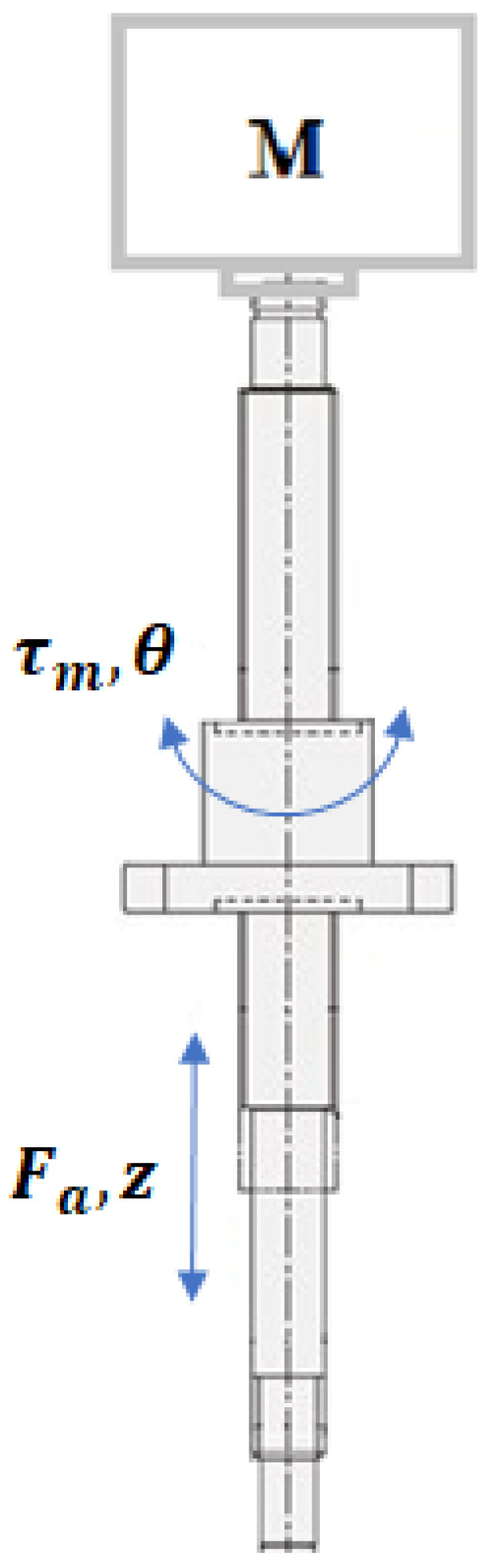
Subsystem assembly motor-ball-screw.

A ball-screw is a mechanical linear actuator that translates rotational motion to linear motion with little friction. A threaded shaft provides a helical raceway for ball bearings which act as a precision screw. As well as being able to apply or withstand high thrust loads, they can do so with minimum internal friction. They are made to close tolerances and are therefore suitable for use in situations in which high precision is necessary. The ball assembly acts as the nut while the threaded shaft is the screw. In contrast to conventional lead-screws, ball-screws tend to be rather bulky, due to the need of a mechanism to re-circulate the balls. In Figure 2, a ball-screw assembly is shown.

As expected, we now have a highly coupled system. In what follows, the kinetic equations of the electrical and mechanical systems are depicted.

The motor model where the relationship between current at the generator coil and the moment is

(3) $$\tau_{i}=k_{t}i$$

where *i* is the current, τ is the torque and $k_{t}$ is the motor torque constant. Considering the inertia of the motor $J_{m}$ and the input torque $\tau_{m}$, we have

(4) $$\tau_{m}-\tau_{i}=J_{m}\frac{d^{2}\theta }{dt^{2}}$$

The inertia of the motor is denoted as $J_{m}$, and $\tau_{m}$ is the input torque.

According to Kirchhoff’s voltage law:

(5) $$k_{e}\frac{d\theta }{dt}-L\frac{di}{dt}-Ri=0$$

where *L* is the armature inductance, *R* represents the total resistance between the internal $R_{i}$ and external $R_{e}$ resistances; $k_{e}$ is the counter electromotive voltage constant according to:

(6) $$k_{e}\frac{d\theta }{dt}=U_{emf}$$

where $U_{emf}$ is the counter electromotive force, which depends directly on the velocity change.

Summarizing the above expressions

(7) $$k_{e}\frac{d\theta }{dt}=\frac{L}{k_{t}}[\frac{1}{k_{t}}\frac{d\tau_{m}}{dt}-J_{m}\frac{d^{3}\theta }{dt^{3}}]+\frac{R}{k_{t}}[\tau_{m}-J_{m}\frac{d^{2}\theta }{dt^{2}}]$$

After some algebraic manipulation, the above expression can be expressed in Laplace domain, leading to:

(8) $$T_{m}=J_{m}\Theta s^{2}+C_{m}\Theta s+K_{m}\Theta$$

with parameters $C_{m}=C_{eq}$ and $K_{m}=K_{eq}$ defined as:

(9) $$\begin{array}{ccc} C_{eq} & = & \frac{k_{t}k_{e}R}{R^{2}+L^{2}\omega^{2}} \end{array}$$

(10) $$\begin{array}{ccc} K_{eq} & = & \frac{k_{t}k_{e}L\omega^{2}}{R^{2}+L^{2}\omega^{2}} \end{array}$$

The mechanical system is represented by a ball-screw mechanism, it transforms the linear motion into a rotational following the relationship

(11) $$z_{b}=\frac{\theta l}{2\pi }$$

Equation (8) in time domain leads to:

(12) $$\tau_{m}=J_{m}{\ddot{\theta }}_{m}+C_{m}\dot{\theta }+K_{m}\theta_{m}$$

where *l* is the ratio motion from the ball-screw, and *z* the relative displacement. On the other hand, $\tau_{m}$ is related with the output torque of the ball-screw mechanism.

(13) $$\tau_{m}=lF_{a}$$

For $F_{a}$ the ball-screw force.

As the ball-screw is in series with the motor, the inertias add up to an equivalent mass:

(14) $$m_{eq}=J_{m}+J_{b}$$

Making a change of variables for the rest of the equivalent coefficients:

(15) $$C_{eq}=\frac{k_{t}k_{e}\left(R_{e}+R_{i}\right)}{{\left(R_{e}+R_{i}\right)}^{2}+L^{2}\omega^{2}}$$

(16) $$K_{eq}=\frac{k_{t}k_{e}L\omega^{2}}{{\left(R_{e}+R_{i}\right)}^{2}+L_{i}^{2}\omega^{2}}$$

**Figure 3:**
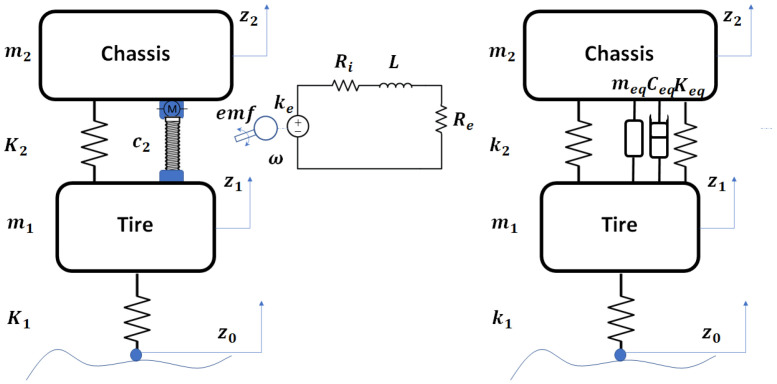
Breakdown of the EHSA system into equivalent parameters $m_{eq}$, $c_{eq}$ and $k_{eq}$.

The EHSA ball-screw acts as a shock absorber where an equivalent damping ($C_{eq}$) is reached, providing an equivalent mass ($m_{eq}$) and stiffness ($K_{eq}$) to the system as shown in Figure 3.

The shock absorber between sprung and unsprung masses is modeled as an electric generator that includes an external resistance, ball-screw, and linear spring. The damping force related to electric resistance and the input excitation to the system *z* were obtained, $\dot{z}$ and $\ddot{z}$ represent the velocity and acceleration input, respectively.

(17) $$\begin{array}{ccc} z & = & -Z_{m}\cos\left(\omega t+\phi \right) \end{array}$$

(18) $$\begin{array}{ccc} \dot{z} & = & \omega Z_{m}\sin\left(\omega t+\phi \right) \end{array}$$

(19) $$\begin{array}{ccc} \ddot{z} & = & \omega^{2}Z_{m}\cos\left(\omega t+\phi \right) \end{array}$$

where $Z_{m}$ is the amplitude of the input excitation and ϕ is the phase angle.

The force in terms of the input excitation yields:

(20) $$F_{a}=\frac{1}{l^{2}}\left(m_{eq}\ddot{z}+C_{m}\dot{z}+K_{m}z\right)$$

Thus, the reacting force provided by the ball-screw damper can be expressed depending in the external resistor Re as:

(21) $$F_{a}=\left(J_{m}+J_{b}\right)l^{-2}\ddot{z}+\frac{k_{t}k_{e}\left(R_{e}+R_{i}\right)l^{-2}}{{\left(R_{e}+R_{i}\right)}^{2}+L^{2}\omega^{2}}\dot{z}+\frac{k_{t}k_{e}L\omega^{2}l^{-2}}{{\left(R_{e}+R_{i}\right)}^{2}+L^{2}\omega^{2}}z$$

In the following section, it is explored how well the ball-screw damper can mimic the behavior of a real damper unit by controlling the external resistance.

## 3. Emulation of Commercial Damper

The purpose of this section is to find an optimal configuration of the system that allows us to emulate the reaction force provided by a real damper, which is used as baseline. This real damper is the one used in the Renault–Twizy electric car, manufactured in Valladolid, Spain, and was tested in a damper test machine obtaining its characteristic force–velocity curve.

The study is focused on finding optimal values of the external resistance, which allow us to obtain a force–velocity curve as close as possible to that of the baseline model. So, in the next section, different electrical approaches can be explored using these optimal values of the external resistance as input command in order to mimic the baseline damper’s response.

Initially, the impact of the different components involved in the reaction force provided by the ball-screw damper is analyzed. These are the stiffness, damping and inertia components corresponding to the terms in Equation (21) with *z*, $\dot{z}$ and $\ddot{z}$, respectively.

The force values produced by this system can be compared to those force values experimentally obtained for the physical (baseline) damper. The parameters corresponding to the ball-screw damper are shown in Table 1 and were obtained according to [16]. All of them, except the load resistor ($R_{e}$), are constant coefficients that correspond to the constructive properties of the system. The load resistor can be varied in order to obtain different behaviors of the system. A behavior close to the baseline damper is obtained for a value of $R_{e}=10$Ω.

### 3.1. Effect of the Force Components on the Ball-Screw Damper Response

**Figure 4:**
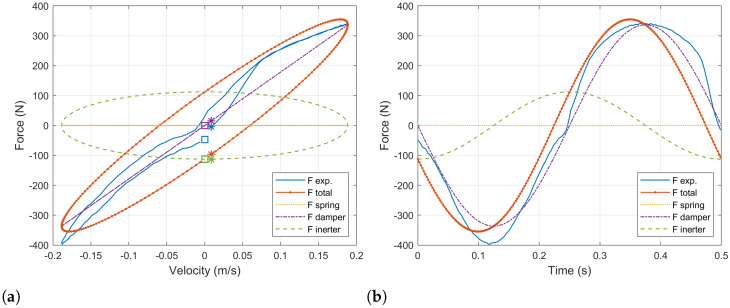
(**a**) Force–velocity and (**b**) force–time diagrams of the ball-screw damper with a constant external resistance $R_{e}=10\;\Omega$ compared to the experimental data of the baseline damper. The ball-screw damper’s force is decomposed into its components corresponding to the equivalent sprint, damper and inerter.

Figure 4a presents with a blue line the force – velocity diagram for the physical damper. Overlapping it, a similar diagram for a simulation of the ball-screw damper with a constant value of the load resistor $R_{e}=10$Ω is shown with an orange dotted line. Additionally, this force is decomposed into the three components involved: the equivalent spring force is plotted in a yellow line, the equivalent damper force is plotted in a purple line and the equivalent inerter force is plotted in a green line. In order to provide a clearer view, the initial points are marked with a square whilst the final points are marked with an asterisk. So that it is possible to see the evolution of the forces and the velocity with time. Additionally, the equivalent force–time diagram is shown in Figure 4b to complement the understanding of the effects of the different force’s components.

It has to be noted that the equivalent spring force in the force–velocity diagram is also an ellipse whose principal axis corresponds to the *x* axis. Nevertheless, it is seen as a straight line because the equivalent spring forces is small compared to the other components and, thus, its secondary axis is much smaller than the principal one. As it can be appreciated, for the ball-screw damper, the equivalent inertia force component predominates at low velocity. This fact will be determinant when one tries to reproduce the response of the physical damper.

The evolution in time of the force can be analyzed in Figure 4b. The initial velocity is zero, which corresponds to an extreme position in the damper’s tip traveling. As the damper is compressed, the velocity is increased up to the middle point in the damper traveling, when it becomes maximum. At this point, the damping component of the force becomes maximum. The acceleration and the inerter components become zero. After this point, the velocity starts to decrease until the damper is completely compressed reaching the other extreme position in the damper traveling. The process is repeated in the opposite sense.

### 3.2. Optimal Load Resistor

As it has been shown in previous section, the equivalent damping ($C_{eq}$) and stiffness ($K_{eq}$) components of the reacting force in the ball-screw damper model depend on the load resistor. This resistor can be controlled to obtain different values of the force depending on the velocity and the position. In order to obtain those values of the load resistor that reproduce the behavior of the baseline damper, an optimization process has been carried out taking advantage of the optimization Matlab’s toolbox.

The optimization problem is defined independently for each sampling time of the experimental data as:

(22) $$R_{opt}=\arg\min_{R_{e}}\left(F_{exp}-F_{a}\left(R_{e},z,\dot{z},\ddot{z}\right)\right)$$

with

(23) $$0\;\Omega <R_{e}<100\;\Omega$$

**Figure 5:**
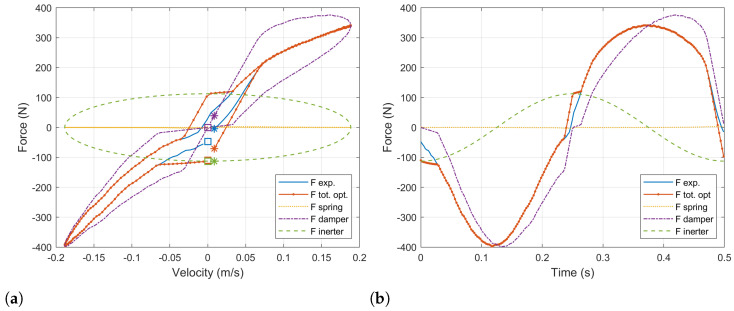
(**a**) Force–velocity and (**b**) force–time diagrams of the ball-screw damper with optimized external resistor ($R_{e}$) compared to the experimental data of the baseline damper. The ball-screw damper’s force is decomposed into its components corresponding to the equivalent sprint, damper and inerter.

Note that the the resistance value is lower and upper bounded. On one hand, this value cannot be negative. On the other hand, the optimization algorithm will try to achieve its target by increasing the value of the external resistance even in those cases in which the target is unachievable. Which will yield an infinite resistance’s values. The upper limit established was observed to return reasonable results, which will be shown and explained below in Figure 5a,b.

For each state of the system, it is found the best value of the load resistor ($R_{e}$) that allows the ball-screw damper’s force $F_{a}$ (21) to emulate the force produced by the baseline damper ($F_{exp}$) at these position, velocity and acceleration conditions. The states under consideration are those corresponding to the force–velocity test carried out on the baseline damper, which means that the motion of both, baseline and ball-screw damper correspond to a sinusoidal signal. The cost function is defined as the signed difference between the baseline damper’s force and the ball-screw damper’s force at each point of the dampers travel. For each of these points, the position, velocity and acceleration are computed. They are passed as parameters to the cost function along with the experimental value of force obtained for the baseline damper at this point.

The resultant force–velocity diagram after this optimization process is shown in Figure 5a. The experimental data of the baseline damper’s force is plotted in a solid blue line. The force computed for the ball-screw damper with the optimal load resistor is plotted with a dotted orange line. The equivalent stiffness, damping and inertia components in which this force can be decomposed are plotted in yellow, purple and green, respectively. Similar results are shown in a force–time diagram in Figure 5b.

At high values of velocity, the behavior of the baseline damper can be precisely reproduced. However, there exist some situations for which this cannot be done. This happens at low velocity when the acceleration and velocity have the same sign and the experimental target force is lower than the equivalent inerter force. In these cases, the equivalent damping force cannot be used to counteract the equivalent inerter force, and the minimum force obtained is that corresponding to the inertia. It can be observed in Figure 5b for times between t=0 s and t=0.025 s and between t=0.24 s and t=0.26 s.

For these cases, the optimization algorithm tries to minimize the difference between the experimental force of the baseline damper and the computed force of the ball-screw damper by increasing the value of the load resistor. These values tend to be infinite so an upper limit is set.

**Figure 6:**
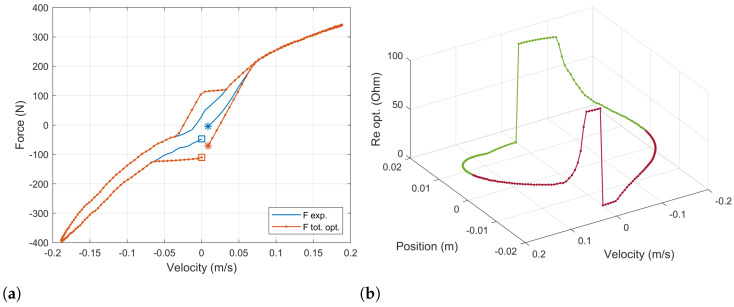
(**a**) Final comparison of the force–velocity diagrams for the optimized ball-screw damper the baseline damper. (**b**) Optimal values of the external load resistor ($R_{e}$) obtained to emulate the baseline damper’s response for the sinusoidal input test.

For the sake of simplicity, Figure 6a shows the force–velocity diagram comparing the experimental data of the baseline damper with the computed force of the ball-screw-damper’s equations. These values were obtained with the optimal values of the load resistor, which are represented in Figure 6b in a 3D plot to show the dependency on both the damper’s position and velocity. As it has been stated, the load resistor ($R_{e}$) is bounded between 0 Ω and 100 Ω.

A first result is that the response of the ball-screw damper is fully controllable through the load resistor in those working conditions for which the damping component of the force is able to counteract the inertia component. However, depending on the internal inertia of the system, there will exist some situations for which the resultant force cannot be set as desired and it will be driven by the equivalent inerter component. This aspect has to be observed in the design of such dampers reducing the inertia of the system in order to obtain better setting capabilities. Nevertheless, the results presented in Figure 4a and Figure 5b correspond to a sinusoidal input in the damper tips, which always implies a phase delay of 90 degrees between the acceleration and the velocity. For real world conditions, bump inputs in the tires do not always present such delay, so the controllability at low velocities could be improved.

**Figure 7:**
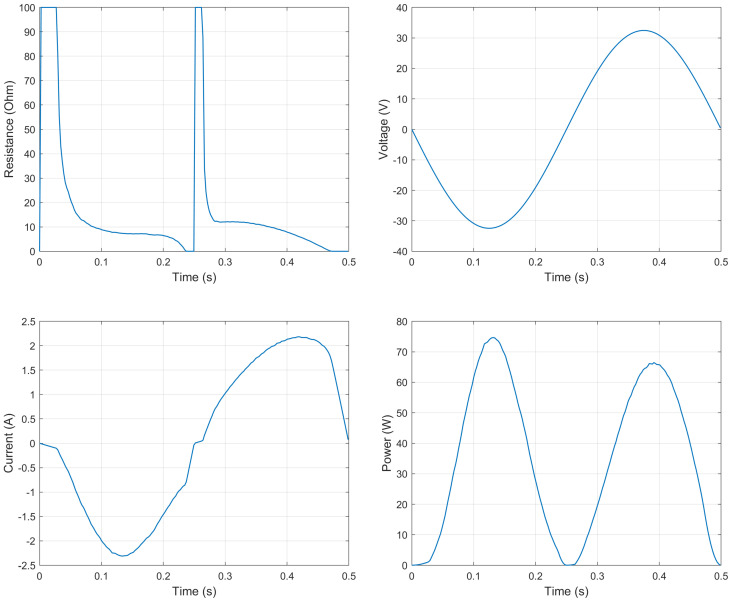
Values of the external resistance, generated voltage, current and power dissipated along one test cycle.

**Figure 8:**
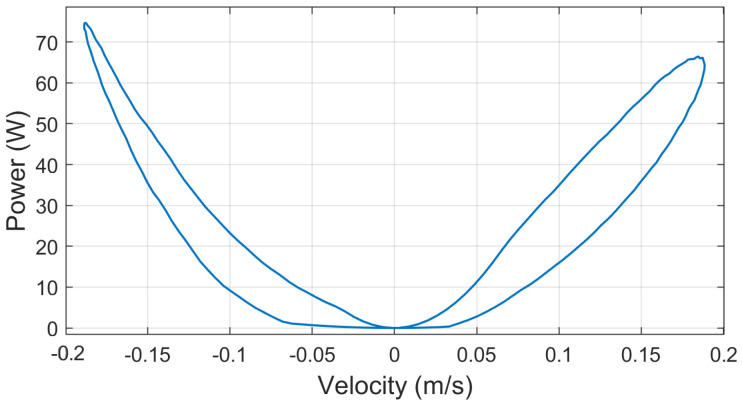
Power dissipated on the external resistor depending in the ball-screw damper’s velocity.

Although the part of the research presented in this manuscript is not focused in the energy recovery part of the problem, the power produced by the system during a test cycle can be estimated by means of the voltage produced at the generator’s terminals and the optimal external resistor’s value at each point of the simulation. Figure 7 presents the values of external resistance, voltage in the generator’s terminal, current flowing through the external resistor and finally the dissipated power for a test cycle. The test cycle is performed at 2 Hz with a peak to peak amplitude of 0.03 m. Under these conditions, the average dissipated power is about 35 W, with peaks of about 74 W for a maximum velocity of 0.19 m/s. Figure 8 represents the dissipated power depending on the velocity.

This results are presented as an estimation of the maximum theoretical power that could be recovered from such a system under this conditions. Nevertheless, as it has been stated before, the study of the energy recovery capabilities of the system is out of the scope of the present research and will be addressed in future works.

The work presented in this manuscript studies the capability of the system to mimic the behavior of a real damper unit. In the next section, the optimal values obtained for the external resistance are used as reference inputs to explore different approaches that may allow achieving the optimal result.

## 4. Implementation Strategy

In Section 3, it was shown that the damping force produced by the Ball-Screw Energy Harvester Shock Absorber (BS-EHSA) system can be adjusted through the external resistor $R_{e}$ connected to the generator’s terminals. In order to have a system that mimics the behavior of a conventional damper, it is needed to introduce an electrical circuit to produce a dynamical value for $R_{e}$ as a function of the velocity between shock absorber terminals. In the research presented in this manuscript, we do not consider the energy recovery component of the system (which will be addressed in future research) and we focus on exploring different approaches to dynamically obtain the optimal value of the external resistor.

**Figure 9:**
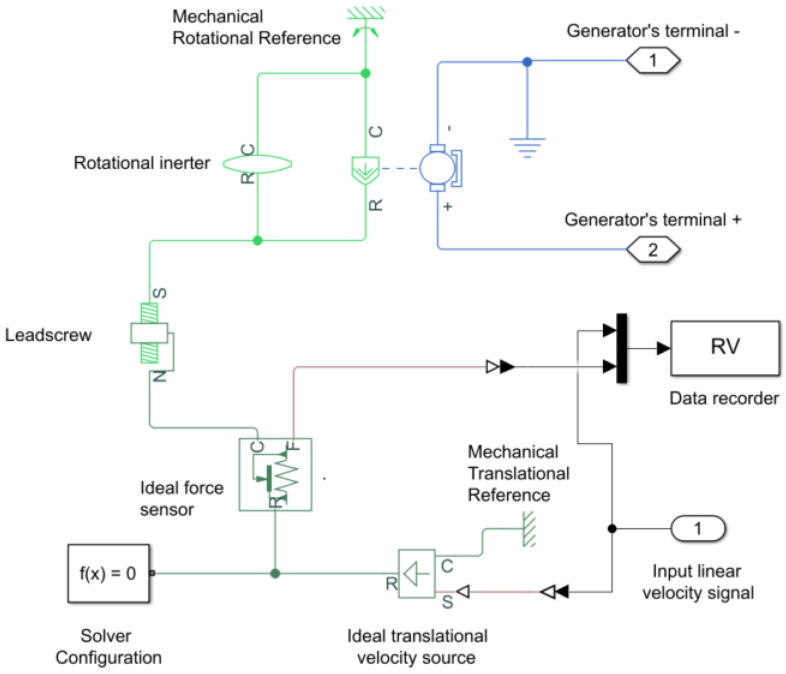
Simscape model for a ball-screw shock absorber.

In Figure 9, the Simscape model used to evaluate different electric-load circuits is presented. The input for our model is the linear velocity signal that is used as a reference for an ideal translational velocity source. This linear movement is transformed into a rotational movement in the leadscrew element that is mechanically connected to the DC motor shaft. The rotational inerter takes into account the rotational mass for the leadscrew system. Finally, the outputs of our model are the electric terminals of the DC-motor where the electric load is connected.

**Figure 10:**
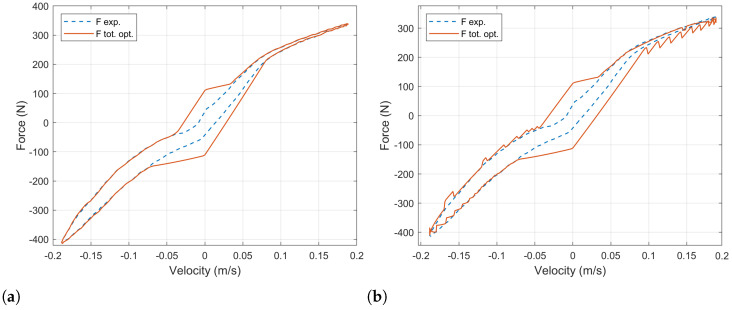
(**a**) Dynamic adjustment of load resistance using an analog potentiometer. (**b**) Dynamic adjustment of load resistance using a digital potentiometer with 40 values.

Figure 10a shows the simulation result for the BS-EHSA system when an analog potentiometer is used to control the force produced by the damper. Its value was adjusted according to Figure 9 by measuring the magnitude and sign of the velocity and acceleration between shock absorber terminals. The blue-dashed line corresponds to the experimental data of the commercial damper analyzed in the previous section, and the blue-dashed line corresponds to the BS-EHSA system with the analog potentiometer. As expected, the BS-EHSA system can mimic the commercial damper behavior for velocities where the electromagnetic force produced at the generator can overcome the inertia in the system. When the velocity is low, it is not possible to adjust the electric torque unless an extra mechanism for current injection is considered. Even though these results are good enough to mimic the behavior of a commercial damper, its implementation using an analog potentiometer implies the design of a servo-controlled system that increases the complexity.

A more straightforward solution can be obtain if a digital potentiometer is used. Figure 10b shows the results when a digital potentiometer with 40 values is used to control the damping force. Blue-dashed line shows small jumps caused by the change between finite values of the resistance. This behavior can cause comfort and stability problems.

**Figure 11:**
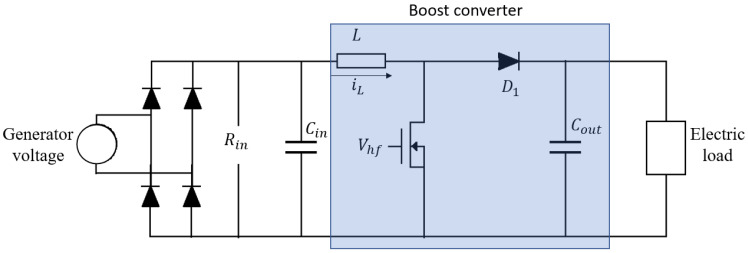
Boost converter as a resistor emulator.

Another approach to change the damping force in the BS-EHSA system is to use a DC–DC converter to emulate a resistor at the input port [31]. This emulation technique is usually employed in power-factor correction applications where a maximum power transfer is required [32,33,34]. In order to evaluate the behavior of the BS-EHSA system using this kind of load, a DC–DC boost circuit has been selected considering the low output voltage at the generator’s terminal for low velocities, and such a circuit is depicted in Figure 11.

**Figure 12:**
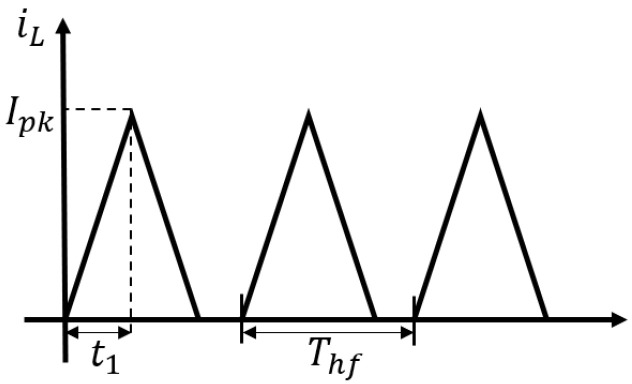
Inductor current.

The voltage generated by the BS-EHSA must be previously rectified to be fed into the DC–DC converter. Then, the current trough the inductor can be adjusted by controlling the switching period for the MOSFET. Figure 12 shows the inductor’s current $i_{L}$ when the converter is running in a Discontinuous Conduction Mode (DCM). As is depicted in Figure 12, in this mode the current through the inductor is equal to 0 for a range of time at the end of the period $T_{hf}$. The corresponding emulated resistance at input port is given by

(24) $$R_{em}=\frac{V_{in}^{2}}{P_{in}}=\frac{2LT_{hf}}{t_{1}^{2}k}\left(\frac{M-1}{M}\right)$$

where $M=V_{o}/V_{in}=1/\left(1-D\right)$ corresponds to the conversion ratio, $t_{1}=DT_{hf}$ is the switching period, *D* is the high-frequency pulse duty cycle ranging from 0 to 1, *L* is the inductance and *k* is the low-frequency pulse duty cycle that considers a periodic shutdown if required, otherwise, *k* is equal to 1 (see [33] for details). Considering k=1, (24) can be simplified as:

(25) $$R_{em}=\frac{2L}{T_{hf}}$$

**Figure 13:**
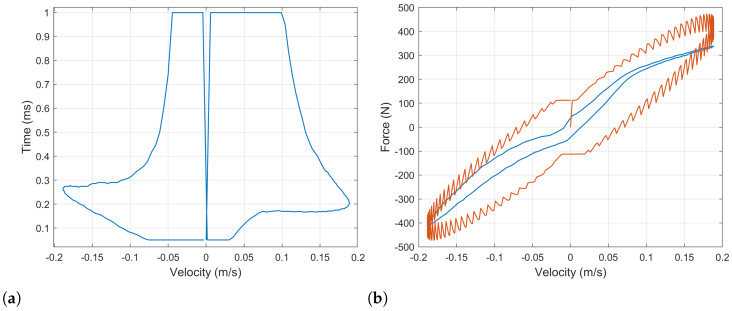
(**a**) DC–DC converter as dynamic load. Closing period as a function of the velocity. (**b**) DC–DC converter as dynamic load. Damping force produced by the BS-EHSA system.

Equation (25) can be used to compute the switching period, taking as an input the resistance shown in Figure 9. In Figure 13a, we have computed the corresponding switching period considering an inductor of 1 mH and an operating frequency for the DC–DC boost of 200 Hz. In the simulation, we have selected $C_{m}=1\;\mathrm{mF}$, $C_{out}=1\;\mu F$ and the electric load, $R_{load}=100\;\Omega$. In Figure 13b, the corresponding BS-EHSA behavior is presented. This result indicates that the effective resistance produce by the DC–DC converter cannot update its value as fast as is required. Even more, its behavior strongly depends on the load element placed in the output, see Figure 11.

**Figure 14:**
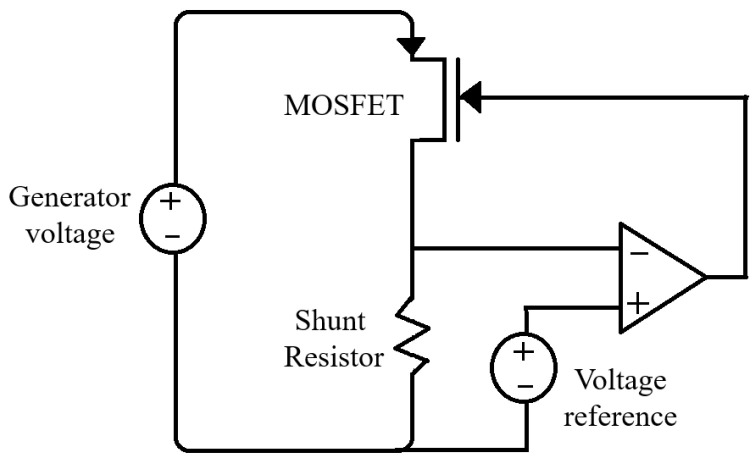
Electronic load circuit in constant current operation.

A better result can be obtained if a simple electric load is used to control the current flowing in the generator’s stator. The electric circuit is shown in Figure 14. The circuit uses a MOSFET to control the current taken from the voltage source. The shunt-resistor is used to measure the current that is compared with the a reference in the operational amplifier.

**Figure 15:**
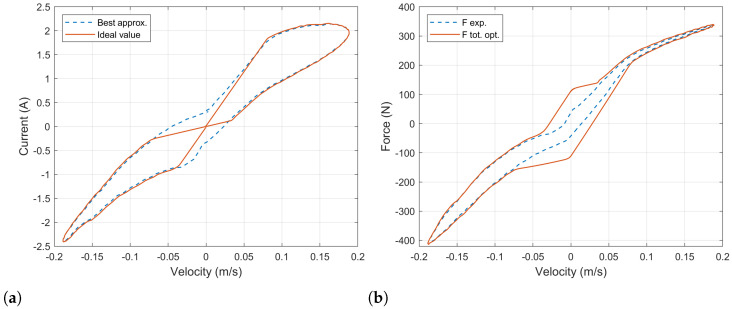
(**a**) DC–DC converter as dynamic load. Closing period as a function of the velocity. (**b**) DC–DC converter as dynamic load. Damping force produced by the BS-EHSA system.

In Figure 15a, the current value used as a reference in the electronic load is presented. The blue-dashed line corresponds to the ideal value that mimics the behavior of a commercial damper. The blue-dashed line shows the best approximation that can be obtained considering the relation between the voltage in the generator and the velocity of expansion/compression in the damper. Finally, Figure 15b shows the damping force produced by the BS-EHSA using an electronic load.

The results show that the BS-EHSA system can produce a very similar curve as the commercial damper when using an electronic load. Again, when the velocity is low, the electromagnetic force cannot overcome the mechanic inertia. In this region, a controlled current source is needed in order to recreate the exact behavior.

## 5. Conclusions

Designing a vehicle shock absorber is based upon desired dynamics and comfort specifications, given a commercial design of a shock absorber, an interesting question is if the dynamical behavior can be reproduced by using a semi-active suspension, particularly an Energy Harvester Shock Absorber (EHSA). This paper studies the possibility to mimic a given shock absorber damping curve.

We considered the commercial damper of the Renault–Twizy electric car as the baseline dynamical behavior and a ball-screw EHSA to mimic its behavior. Force–velocity curves were compared of both, the baseline damper and the BS-EHSA with acceptable results within a certain region.

Different methods for controlling a regenerative damper through the variation of a load resistance are studied to prioritize the movement replication. In this way, the dynamic behavior of the regenerative damper can be matched to the original damper of interest, while recovering part of the wasted energy.

We have evaluated four different approaches to dynamically change the current in the BS-EHSA system in order to mimic the behavior of a commercial damper. An analog potentiometer produces excellent results; however, its implementation leads to mechanical challenges that are difficult to overcome. A digital potentiometer can be easily implemented but the BS-EHSA damper shows undesired “jumps” during the expansion and compression movements (see Figure 10b). This behavior can be attributed to the finite number of resistor values. In order to achieve a continuous range of the electric load, a DC–DC converter was tested; although, this circuit can be easily implemented and its resistance input-value can be continuously change in a predefined range, the resistance rate-of-change is too slow and the BS-EHSA damper cannot follow the reference force curve. The best results were obtained using the electrical load circuit based on a MOSFET. This circuit uses the desired current in the generator stator as a reference to adjust the resistance in the MOSFET’s Chanel. A very good recreation of the force curve (see Figure 13b) of the commercial damper was obtained, with the exception of low velocities, where the electromagnetic force cannot overcome the mechanical inertia.

Finally, our study demonstrates that a given damping curve can be successfully mimicked whenever the velocity of the suspension is high enough, as is shown in Figure 15b. For close-to-zero velocities, the inertia of the system prevents the system from achieving a suitable dynamic behavior unless external energy is deployed.

Future research will be done to analyze different energy recovery circuits applicable to the EHSA system compatible with the result presented here, prioritizing firstly the suspension characteristics and then optimizing the energy harvesting.

## Figures and Tables

**Table 1:** Parameters for the ball-screw damper.

Parameter	Value	Units
Excitation frequency *f*	2	Hz
DC motor EMF, torque constant $k_{e}$, $k_{t}$	0.137	Vs/rad, Nm/A
Internal resistance of DC motor $R_{i}$	6.6	Ω
External resistance $R_{e}$	10	Ω
Inductance *L*	1.7	mH
Motor inertia $J_{m}$	$121\times 10^{-7}$	Kg m^2^
Ball-screw inertia $J_{b}$	$180\times 10^{-7}$	Kg m^2^
Ball-screw lead *l*	5	mm
Amplitude $Z_{m}$	0.015	m

## Data Availability

Not applicable.
